# Influence of Starch Composition and Molecular Weight on Physicochemical Properties of Biodegradable Films

**DOI:** 10.3390/polym11071084

**Published:** 2019-06-26

**Authors:** Daniel Domene-López, Juan Carlos García-Quesada, Ignacio Martin-Gullon, Mercedes G. Montalbán

**Affiliations:** Chemical Engineering Department, University of Alicante, Apartado 99, 03080 Alicante, Spain

**Keywords:** TPS film, amylose content, molecular weight, crystallinity, mechanical properties

## Abstract

Thermoplastic starch (TPS) films are considered one of the most promising alternatives for replacing synthetic polymers in the packaging field due to the starch biodegradability, low cost, and abundant availability. However, starch granule composition, expressed in terms of amylose content and phosphate monoesters, and molecular weight of starch clearly affects some film properties. In this contribution, biodegradable TPS films made from potato, corn, wheat, and rice starch were prepared using the casting technique. The effect of the grain structure of each starch on microstructure, transparency, hydration properties, crystallinity, and mechanical properties of the films, was evaluated. Potato starch films were the most transparent and corn starch films the most opaque. All the films had homogeneous internal structures—highly amorphous and with no pores, both of which point to a good starch gelatinization process. The maximum tensile strength (4.48–8.14 MPa), elongation at break (35.41–100.34%), and Young’s modulus (116.42–294.98 MPa) of the TPS films were clearly influenced by the amylose content, molecular weight, and crystallinity of the film. In this respect, wheat and corn starch films, are the most resistant and least stretchable, while rice starch films are the most extensible but least resistant. These findings show that all the studied starches can be considered suitable for manufacturing resistant and flexible films with similar properties to those of synthetic low-density polyethylene (LDPE), by a simple and environmentally-friendly process.

## 1. Introduction

Approximately 40% of food packaging is made of plastic materials [1]. During the last decades, the use of petroleum-based plastics such as low-density polyethylene (LDPE) for this purpose has been highly intensive. However, due to dwindling petroleum resources, the extensive use of one-use packaging materials, and the severe pollution problems related with their recycling, the development of renewable and environmentally-friendly polymers has become a priority concern [2,3,4]. In an attempt to solve this problem, biopolymers such as starch and its derivatives (polylactic acid, etc.), cellulose, collagen, and chitosan, have been considered as alternatives because they are abundant, inexpensive, and biodegradable [1,3,5,6]. Of these, starch is probably the most promising option because it can be readily cast into thermoplastic starch (TPS) films, which are odorless, colorless, transparent, and of very low oxygen permeability [7]. In addition, starch is widely produced in industry, which is an advantage for its use in food packaging [8]. Starch is a carbohydrate polymer composed of two macromolecules: amylose, a linear polysaccharide with an average molecular mass of 10^5^ g·mol^−1^; and amylopectin, a branching polysaccharide with an average molecular mass of 10^6^–10^7^ g·mol^−1^. Hydrogen bonds hold the starch chains together, making it insoluble in cold water. In order to obtain a TPS film, starch granules must be gelatinized with a plasticizer in an excess of water at temperatures lower than 100 °C. Commonly used plasticizers are monosaccharides, disaccharides, oligosaccharides (glucose, fructose), and polyols (glycerol, sorbitol). In this way, hydrogen bonds are formed between the plasticizer and the starch in an irreversible gelatinization process. 

Starch can be obtained from a great variety of botanical species, which determines the compound’s grain size, size distribution, shape, amylose/amylopectin ratio, molecular weight, and phosphate monoester content. Corn, wheat, potato, and rice starch are the most common starch sources, representing 84%, 7%, 4%, and 1% of world production, respectively [9]. The starches have different film-forming properties, and the chemical, physical, and mechanical properties of the TPS films obtained from them may vary widely [8]. Few studies have compared the properties and structures of TPS films obtained by casting using different starches [8,9,10] and discrepancies exist with regard to the tensile properties, microstructure, and degree of plasticization of the films. To the best of our knowledge, this is the first time that a systematic comparative study has been made of starch grain characterization and of the microstructure, crystallinity, and mechanical properties of the TPS films obtained from potato, corn, wheat, and rice starch. In addition, this is the first time that the properties of TPS films obtained from the above-mentioned starches have been correlated with the starch granule composition (amylose and phosphate monoester contents) and molecular weight of the starch grains. This work, therefore, should contribute to a better understanding of the properties of biodegradable TPS films obtained from tuber (potato) and cereal (corn, wheat and rice) starches, thereby contributing to their successful application in the packaging industry. 

The aim of this work has been to produce biodegradable TPS films from four different starch sources (potato, corn, wheat, and rice) by the casting technique, using glycerol as plasticizer (33 wt % on starch basis) and to compare their properties. Three sub-objectives are proposed: (i) to characterize the starch grains in terms of their molecular weight, microstructure, size distribution, amylose, phosphate monoester contents, and humidity; (ii) to characterize the TPS films in terms of their hydration properties, migration into food simulants, microstructure, crystallinity, and tensile properties; and (iii) when possible, to establish possible relationships between the starch granule composition and molecular weight of each starch and the subsequent film properties.

## 2. Materials and Methods

### 2.1. Materials

Corn starch (24.8% amylose, 0.020% phosphorus content, 11% moisture content), wheat starch (24.5% amylose, 0.038% phosphorus content, 9.6% moisture content) and rice starch (16.9% amylose, 0.030% phosphorus content, 9.2% moisture content) were purchased from Sigma-Aldrich (Madrid, Spain). Potato starch (20.5% amylose, 0.043% phosphorus content, 15.1% moisture content) was provided by Across Organics (Geel, Belgium), and the plasticizer glycerol was supplied by Fisher Chemical (Geel, Belgium). All chemicals were used without further purification.

### 2.2. Starch Grains Characterization

The morphology and particle size of the four starches were determined by Scanning Electron Microscopy (SEM) and laser diffraction. The shape of the starch grains was observed by SEM (Hitachi, model S3000N, Tokyo, Japan) with an accelerating potential of 15 kV. The particle size analysis was carried out by laser diffraction (Malvern Instruments, model 2000, Worcestershire, UK). 

The Megazyme amylose/amylopectin assay procedure, using the commercial kit, was followed to quantify the amylose content of the starches. Determining phosphorus content was conducted with an inductively coupled plasma mass spectrometer (ICP-MS, Agilent 7700x, Agilent Technologies, Waldbronn, Germany). Gel Permeation Chromatography (GPC) coupled with Multi Angle Light Scattering (MALS) was used to determine the weight-average molecular weight of the starches. A Size-exclusion Chromatographer (SECurity 1260, Polymer Standard Service, Mainz, Germany) with double detection (MALS and refractive index) was employed. The mobile phase was DMSO with 0.1 M LiCl. A certain amount of each sample (≈20 mg) was exactly weighed and added to a defined volume of mobile phase. After that, the samples were dissolved at 80 °C. All solutions were filtered through a 1 µm filter before being injected into the GPC precolumn (PSS-Suprema, 10 μm, Guard, ID 8.0 mm × 50 mm) and analytical column (PSS-Suprema, 10 μm, 10,000 Å, ID 8.0 mm × 300 mm). The column temperature was controlled at 80 °C and the flow rate was 0.3 mL/min. The weight-average molecular weight of the starch samples was calculated using the WinGPC UniChrom software version 8.3 (Polymer Standard Service, Mainz, Germany).

The moisture content of the starches was determined by their loss of weight after drying in an oven for 5 h at 110 °C. Crystallinity of the starch grains was studied by X-ray diffraction on a Bruker diffractometer (D8-Advance model, Ettlingen, Germany) equipped with a KRISTALLOFLEX K 760-80F X-ray generator (Power = 3,000 W, Voltage = 20–60 kV and Intensity = 5–80 mA) which has an X-ray tube with copper anode (λ = 1.54056 Å). The equipment operated at 40 kV and 40 mA with 2θ varying from 10° to 60° with a step size of 0.05°.

### 2.3. Preparation of TPS Films

Starch films were prepared by the casting technique following the procedure described by Medina-Jaramillo et al. [11]. Briefly, the film-forming solution was composed of potato, corn, wheat or rice starch (5 wt %), glycerol (1.5 wt %), and distilled water (93.5 wt. %). The solution was stirred at room temperature for 45 min and then heated at 96 °C for 40 min to guarantee complete starch gelatinization. The mixture was then cooled, degassed under vacuum for 7 min, and finally poured onto Petri dishes (0.34 g/cm^2^). The plates were dried in an oven with circulating air at 50 °C for 48 h. The dried films were peeled off and stored at room conditions (around 25 °C and 50% relative humidity) for a week before characterization. 

### 2.4. Film Characterization

#### 2.4.1. Film Thickness

Film thickness was measured in different locations with a Palmer Electronic digital micrometer (Madrid, Spain). The mean value was calculated and used for the rest of the calculations. 

#### 2.4.2. Hydration Properties

The water content and solubility in water of the films were determined with samples of 1 × 1 cm^2^ following procedures described in the literature with some modifications [3,11]. Firstly, the water content was measured by determining the weight loss of the films after drying in an oven for 5 h at 110 °C. The measurements were taken in quadruplicate. The quantity of absorbed water or moisture content was expressed as percentage (grams of water in 100 grams of sample) using Equation (1):

(1) $$Water\;content\;\left(\text{\%}\right)=\left(\frac{m_{0}-m_{1}}{m_{0}}\right)\times 100$$

where *m*_0_ and *m*_1_ are the mass before and after drying, respectively.

After that, the water solubility of the samples was measured by placing the above dried films individually in 10 mL tubes filled with 9 mL of distilled water. The tubes were capped and stored at 25 °C for 24 h, after which the films were taken out and dried again at 110 °C for 5 h in order to determine the final mass of dry matter, *m_f_*. Water solubility was calculated from the loss of total soluble matter as follows (Equation (2)):

(2) $$Solubility\;\left(\text{\%}\right)=\;\left(\frac{m_{0}-m_{f}}{m_{0}}\right)\times 100$$

The solubility values were taken as the average of at least four repetitions.

#### 2.4.3. Migration into Food Simulants

Migration studies were carried out following the current legislation [12]. The simulants chosen were: simulant A, ethanol (10% *v*/*v*, simulating hydrophilic foods); simulant B, acetic acid (3% *w*/*v*, simulating acid foods); and simulant C, isooctane (simulating lipophilic foods with free fats at the surface). Film samples were immersed in 9 mL of the simulants with a contact ratio of 6 dm^2^ of film per kg of simulant. In accordance with Test number OM1, the films were left in contact with the food simulants for 10 days at 20 °C, after which the samples were withdrawn, dried and weighed. The difference between the initial and final weight corresponds to the mg of the film components released/dm^2^ of contact surface. The tests were run in quadruplicate.

#### 2.4.4. Transparency

The transparency (in terms of opacity) of the films was determined using a basic VIS V-1200 spectrophotometer (VWR, Barcelona, Spain) at a wavelength of 600 nm following the procedure used by other researchers [1]. The opacity was calculated as shown in Equation (3):

(3) Opacity =Abs600x

where *x* is the thickness (expressed in mm) of the film and *Abs_600_* is the absorbance measured at 600 nm. Lower values of the opacity parameter, as defined on Equation (1), imply greater transparency.

#### 2.4.5. Scanning Electron Microscopy (SEM)

The microstructure of the films was observed from SEM images obtained with a Hitachi Scanning Electron Microscope (Hitachi S3000N, Tokyo, Japan) using an accelerating voltage of 5 kV. The images were taken on the surface and in cross sections of all the studied films. Dried sheet samples were cryofractured after immersion in liquid nitrogen. Before the analysis, the samples were coated with gold for better observation.

#### 2.4.6. Atomic Force Microscopy (AFM)

Film surfaces were also analyzed by an NT-DMT Atomic Force Microscope (NTEGRA Prima model, Moscow, Russia) operating in tapping mode. The images were processed using the software Nova Px (NT-MDT Co., Moscow, Russia, 2013) and three-dimensional images of the film surfaces (10 µm × 10 µm) were obtained. 

#### 2.4.7. Mechanical Properties

The mechanical tensile properties of the films were determined with an Instron 3344 Universal Test instrument (Norwood, MA, USA) equipped with 2000 N load cell and operated at 25 mm/min following ASTM D882-12 (2012) [13] standard recommendations. The samples were cut into dumbbell-shaped specimens. Mechanical properties of each film were calculated using the average thickness of the specimen and at least five specimens per sample were tested. The tensile properties studied were tensile strength at break, elongation at break, and Young’s modulus.

#### 2.4.8. X-ray Diffraction (XRD) Studies

Film diffractograms were recorded on a Bruker diffractometer (D8-Advance model, Ettlingen, Germany) equipped with a KRISTALLOFLEX K 760-80F X-ray generator. The equipment operated at 40 kV and 40 mA, with 2θ varying from 10° to 60° with a step size of 0.05°. The FITYK software (0.9.8 version, Warsaw, Poland) was used for curve fitting. 

## 3. Results and Discussion

### 3.1. Starch Characterization

**Figure 1:**
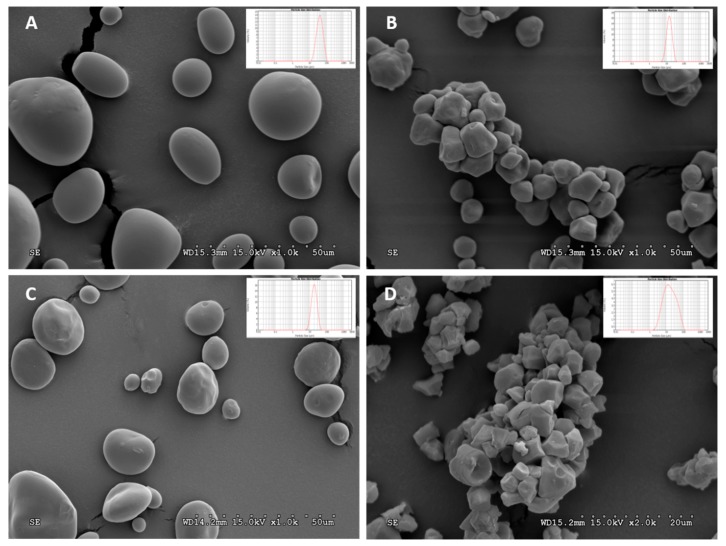
SEM micrographs of (**A**) potato starch (1000×), (**B**) corn starch (1000×), (**C**) wheat starch (500×), and (**D**) rice starch (2000×). Different magnifications were used for a clearer observation of the granules. The particle size distribution profiles, as measured by laser diffraction, are given for each starch.

Figure 1 shows the SEM micrographs of the starch granules obtained from four sources: potato, corn, wheat, and rice. The granule shape is greatly influenced by the starch source, as previously reported [14]. Potato starch granules have an oval shape, corn and rice starch granules have a polyhedral shape, and wheat starch granules have a lenticular shape. Their general appearance is similar to that previously mentioned by other researchers [8,14,15,16]. The micrographs point to different size distributions, as confirmed by laser diffraction measurements.

Starch granule size was determined by laser diffraction and the results are shown on Table 1. Potato starch presented the highest grain size ranging from 24 to 73 µm. Corn and wheat starches presented similar average diameters and size distributions ranging from 10 to 24 µm and 12 to 31 µm, respectively. Particle size analysis of rice starch ranged from 5 to 52 µm and showed wider size dispersion, as can be deduced from the higher span value and standard deviations. The average diameter of the starch grains was 46.02, 16.38, 20.75, and 23.30 µm for potato, corn, wheat, and rice, respectively. These results are in good agreement with those obtained by SEM, which pointed to the higher average diameter of potato starch grains with respect to others. In addition, rice starch seems to have a wider size distribution and great agglomerations of grains, which might explain the higher span value found.

The amylose content of the starches was similar for all the starches although with slight differences. These differences could have certain influence on the different properties obtained for the films, as it will be discussed later. In order, the amylose content of the starches was the following: rice (16.9%) < potato (20.5%) < wheat (24.5%) ≈ corn (24.8%). The literature reports considerable discrepancies in the amylose content of starches, mainly due to the exact botanical origin of each starch and the determination method. Nevertheless, the amylose content of the starches we recorded and the values presented in the literature were in good agreement [9,17,18,19].

In contrast to amylose content, the values of weight-average molecular weight of the studied starches have shown significant differences. We found that the molecular weight of rice starch is the highest reaching a value of 83.2 MDa. Potato starch presents an intermediate value (69.5 MDa) and corn and wheat starches have similar and the lowest values (≈51 MDa). The origin of these values could be in the molecular weight of the two macromolecular constituents of starch, amylose, and amylopectin. As stated in the *Introduction* Section, amylose is a linear polysaccharide with an average molecular mass of 10^5^ g·mol^−1^ and amylopectin is a branched polysaccharide with an average molecular mass of 10^6^–10^7^ g·mol^−1^. For this reason, starches with high amylose content could presumably have lower molecular weight and a relatively more linear structure than those with a high content of amylopectin. 

The amount of phosphate monoesters contained in starch is usually very low and depends on plant origin. Generally, potato starch contains a higher phosphate monoester quantity than cereal starches [20,21]. As expected, in this work the phosphorus content of potato starch was the highest as can be seen in *Materials* Section. In starch, the phosphorus is present in the form of phosphate monoester [22]. Therefore, potato starch contains the highest value of phosphate monoester. 

With respect to the moisture content of the starches (determined by a gravimetric method), the most hygroscopic starch was that obtained from potato, which reached values of around 15%, while the other starches showed similar values of around 9–11%. This difference could be related to the fact that potato starch is more phosphorylated and hence has a significant higher hydration capacity [23]. All the values are in good agreement with those reported in the literature [8,14].

### 3.2. Characterization of Starch-Based Films

**Figure 2:**
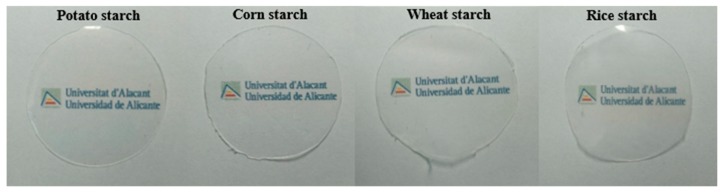
Visual aspect of films obtained from potato, corn, wheat, and rice starch.

Figure 2 shows the visual appearance of the TPS-based films synthesized from potato, corn, wheat, and rice. At a macroscopic scale, all the films were transparent, homogeneous, and easy to handle. The thickness of the films ranged between 0.183 and 0.222 mm (see Table 2). As can be observed, the standard deviation values for the thickness were small, underlining the uniformity of the films obtained. 

#### 3.2.1. Hydration Properties

Table 2 shows the results obtained for the water content and water solubility of the films. With regard to the application of the synthesized films for food packaging, water content of the films is of considerable importance because both starch and food are highly hygroscopic. In addition, water acts as a plasticizer of starch so the presence of water molecules in films could alter their properties [24]. The films obtained from wheat and rice starch showed similar water content values of ~12%. Potato starch had a slightly higher water content (~14%) and corn starch had the lowest value. The water content of the obtained films was considerably lower than the values reported by Basiak et al. [9] and Luchese et al. [8,14], and could represent an interesting advantage for applications involving foodstuffs.

The degree of water solubility of biodegradable films made from water-sensitive biopolymers such as starch is a key parameter, and water solubility is closely related to biodegradability [25]. Water insolubility may be useful for specific applications of films, such as the manufacture of biodegradable packaging, ensuring, among other things, product integrity and water resistance [9], although, for example, edible films for candies require a high degree of water solubility [26]. As shown in Table 2, the water solubility values of the films were around 28–32% for all the samples, which is similar or even lower than the values found in the literature [14,27]. In all cases, the films showed great integrity until the end of the solubility tests. Based on these findings, then, it can be inferred that neither parameter—water content or solubility in water—is greatly influenced by the starch granule composition or molecular weight of the starch.

#### 3.2.2. Migration into Food Simulants

**Figure 3:**
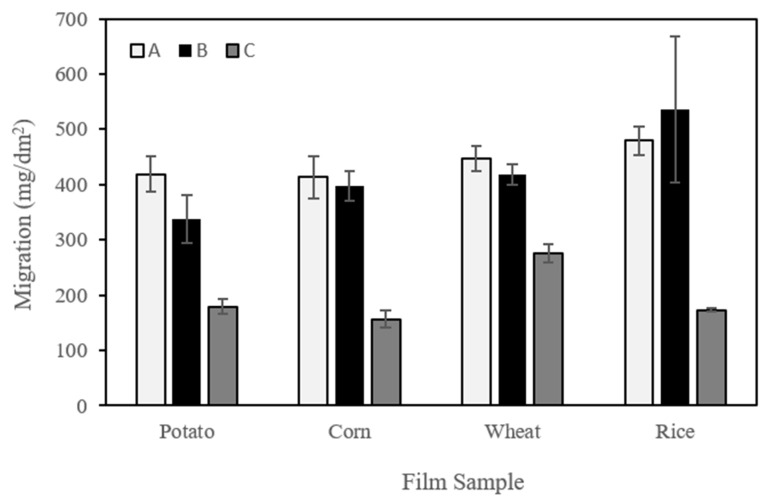
Overall migration values into different food simulants. (**A**) Ethanol (10% *v*/*v*), (**B**) acetic acid (3% *w*/*v*), and (**C**) isooctane.

Figure 3 shows the mean values obtained for the overall migration of the films into food simulants. In our experiment, ethanol 10% *v*/*v* (simulant A) simulated hydrophilic food, acetic acid 3% *w*/*v* (simulant B) acid hydrophilic food (pH under 4.5), and isooctane (simulant C) lipophilic food with free fats at the surface. The three simulants chosen present different polarities, and, in all cases migration. Hence solubility of the components was considerably greater when they were immersed in the hydrophilic simulants (simulants A and B). These results are in good agreement with above mentioned water solubility results since all the films were of a highly hydrophilic nature. Similar results were also obtained in a previous work on corn starch films [28]. A comparison of the films obtained from the different starch sources suggests that there are no significant differences in terms of their migration behavior. 

#### 3.2.3. Transparency

Transparency is a key parameter for films since they are primarily used for food packaging, and a high degree of transparency is usually related to its acceptability as packaging material because of the better visual presentation of the food [26]. However, different degrees of film transparency may be also useful, depending on the specific application. For example, materials with low transparency may help increase the shelf life of some packaged products, and highly transparent films can reduce antimicrobial activity [29]. The transparency results obtained in our experiment are shown on Table 2. Similar or lower transparency values were found by Hornung et al. [3]. The potato starch film was considerably more transparent than the rest of the films, while the corn starch film was the least transparent. The same conclusion was reached by Basiak et al. [9] and Dai et al. [10]. This lower transparency of corn starch films might be due to the high lipid content and yellowish color of corn starch as a result of the presence of small quantities of impurities or pigments [9]. In addition, Hizuruki [30] reported that cereal starches usually contain more short chains and fewer long chains in the amylopectin branches than tuber starches. For this reason, we can assume that amylopectin in potato starch contains a high number of long chains which could contribute to a worst reordering of the chains and the formation of a less compact and hence more transparent starch matrix [19]. In any case, a certain degree of opacity may be suitable for applications in which protection against incident light is important; for example, for wrapping products which can be degraded by light-catalyzed reactions [31]. Nevertheless, at macroscopic scale, these differences in transparency are almost negligible, as can be seen from Figure 2, so the difference in transparency would not affect to the general use of a given type of starch to obtain biodegradable starch films. 

#### 3.2.4. Scanning Electron Microscopy (SEM)

**Figure 4:**
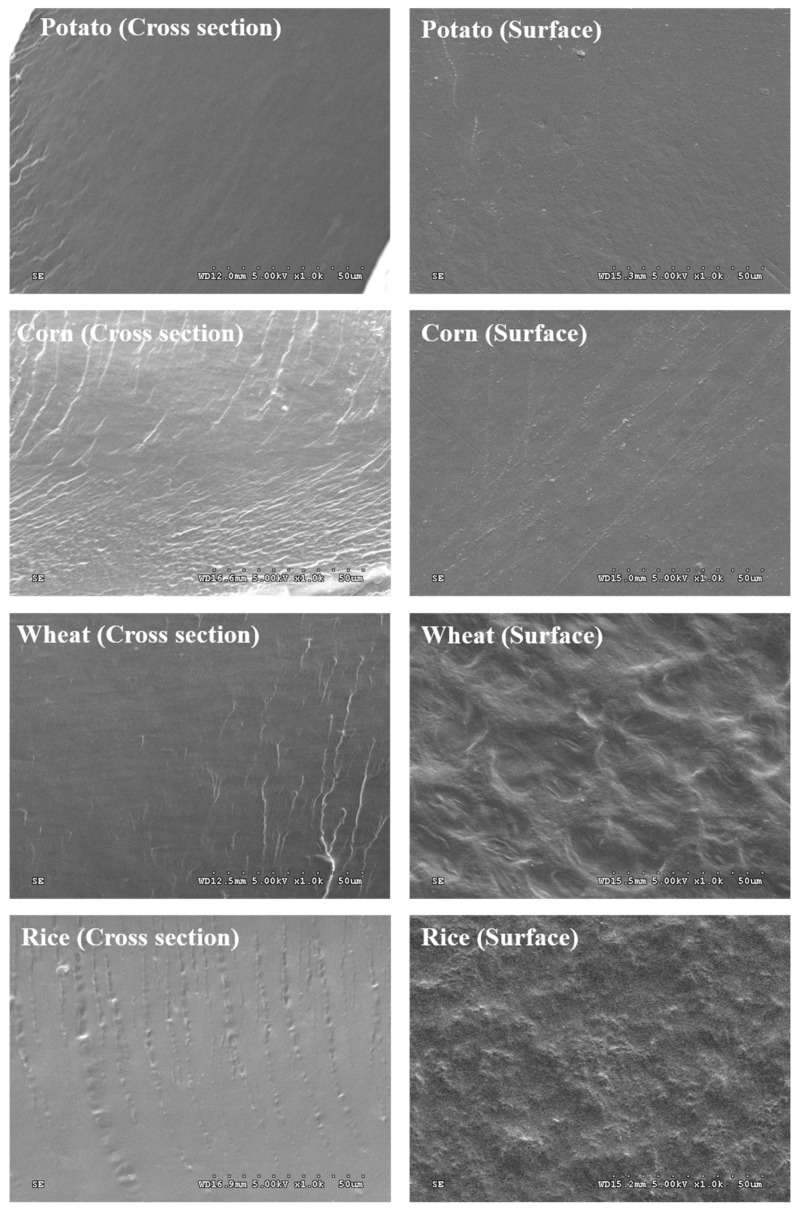
SEM micrographs of the cross sections and surfaces of potato, corn, wheat and rice starch films (Magnification 1000×).

SEM micrographs of the TPS matrix provide interesting information about the microstructure of the obtained films in terms of their homogeneity, layer structure, pores and cracks, and surface smoothness [32]. SEM micrographs of film cross sections and surfaces can be seen in Figure 4. The cross section images for all the starches point to a homogeneous internal structure without pores, reflecting a good starch gelatinization process and the disruption of all the starch granules, as observed by other researchers [7,11]. The absence of defects in the matrix and on the film surface is indicative of the structural integrity of the matrix, which could explain the better tensile strength of the obtained films compared with the findings of previous works, as will be discussed later. It would also explain the good transparency results obtained, along the lines mentioned by Hosseini et al. [33].

#### 3.2.5. Atomic Force Microscopy (AFM)

**Figure 5:**
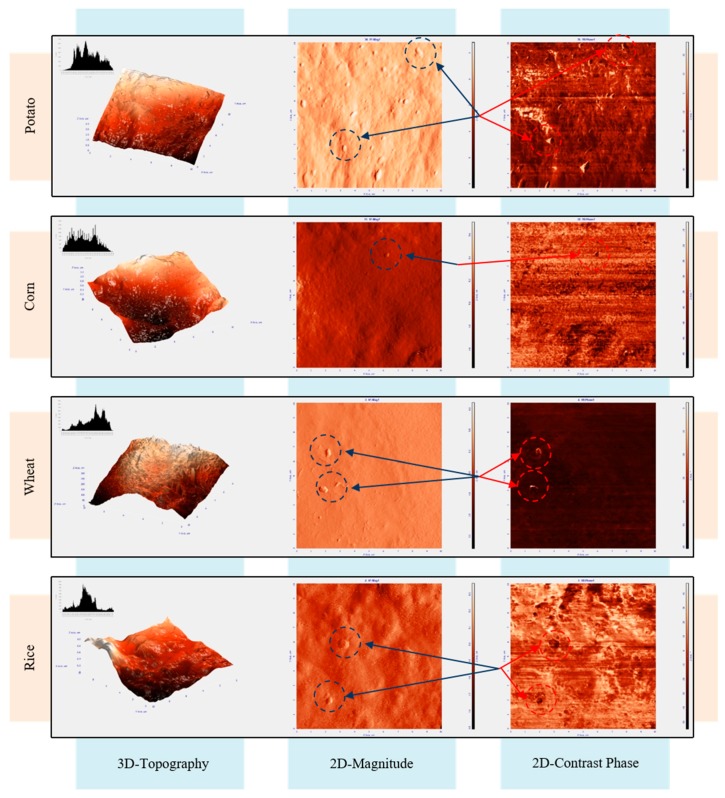
AFM three- and bi-dimensional topographic images and bi-dimensional contrast phase images of the surface of potato, corn, wheat, and rice films.

AFM helps to understand the surface morphology and homogeneity of films by means of a topographic study based on the atomic interaction between constituents [34]. This technique provides qualitative (morphology) information of the studied films. Figure 5 shows tri- and bi-dimensional topographic AFM images of the films, which contribute to the characterization of their surface, and bi-dimensional contrast phase AFM images, which provide information about the existence or absence of phases with different mechanical properties. In our case, the technique allows the identification of the remains of unplasticized starch. Figure 5 points to two different types of behavior. The AFM images of the films obtained from potato, corn and rice starch pointed to a surface with rounded and non-homogeneously dispersed peaks, whereas the topography of the films obtained from wheat starch showed a more homogeneous distribution of sharper peaks. Two-dimensional (2D)-contrast Phase images can contribute to understanding the degree of plasticization of the different films through the observation of darker and lighter regions in the *z*-axis. As expected from the SEM results, all films were very homogeneous without big granule structures. The presence of unplasticized starch in all the starch films was almost missing and the size of the isolated ghost granules observed was much lower than that of the starch grains, indicating the great capacity of glycerol to act as starch plasticizer. Even in the case of potato starch films, which showed a more pronounced presence of starch rests, these granules were less than 2% of the size of the native potato starch grains, pointing to a high degree of plasticization. The small amount of ghost granules observed by AFM could be responsible for the small crystalline diffraction peaks obtained by the XRD analyses, as will be discussed later.

#### 3.2.6. X-ray Diffraction (XRD) Studies

**Figure 6:**
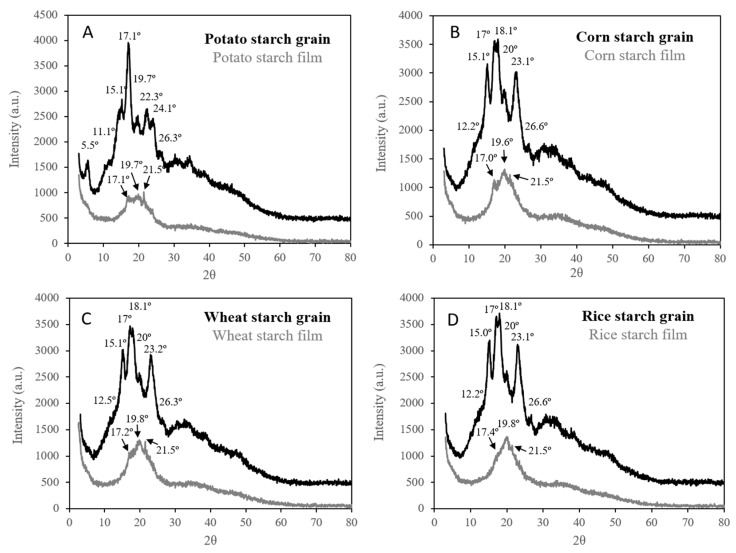
XRD of the starch grains and obtained films.

XRD was used to investigate the crystal structure of the films obtained. The crystalline organization of native starch was seen to be strongly influenced by its botanical origin. Cereal starches such as corn, wheat, or rice starch, typically present an A-type crystal structure, while a B-type structure is characteristic of tuber starches such as potato starch [8,35,36]. Figure 6 shows the XRD patterns of the studied starches and the developed TPS films. On the one hand, native starches from cereals (corn, wheat and rice) showed a very similar pattern, with XRD peaks at 2θ values of 12.2°, 15.1°, 17°, 18.1°, 20°, 23.1°, and 26.6°, indicating A-type crystallinity as previously reported [8,14,35]. On the other hand, potato starch (tuber origin) showed B-type crystallinity, with XRD peaks at 2θ values of 5.5°, 11.1°, 15.1°, 17.1°, 19.7°, 22.3°, 24.1°, and 26.3°, which are in good agreement with the values reported in the literature [8,17,37]. 

**Figure 7:**
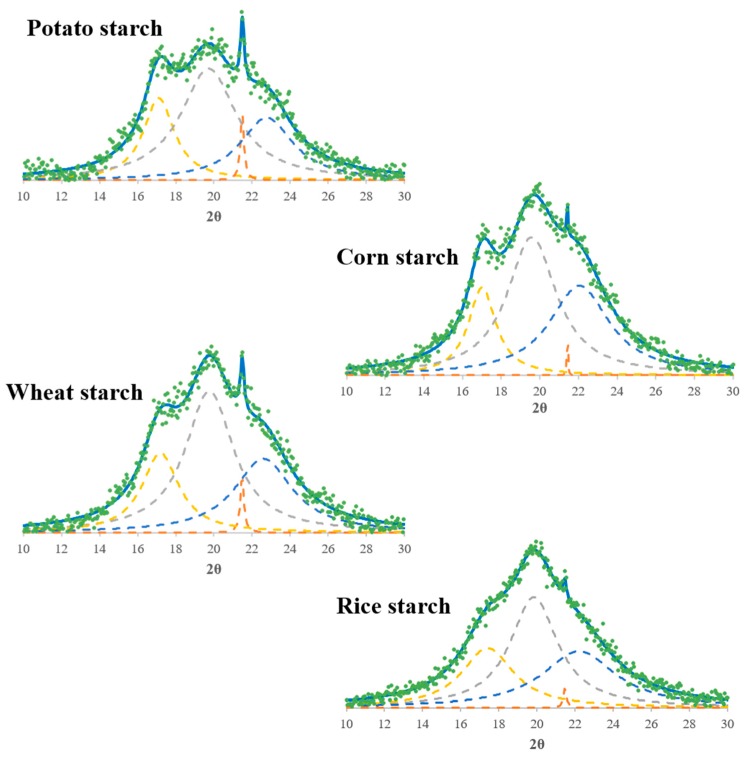
Deconvoluted XRD peaks of starch films obtained from potato, corn, wheat, and rice starch using the Lorentz function.

A loss of crystallinity was expected after the plasticization process to obtain starch films due to disruption of the intermolecular hydrogen bonding between starch molecules by the glycerol molecules, which increases the chain mobility of the starch molecules [38]. A comparison of the XRD patterns of the native starches and of those of the corresponding films confirmed the great loss of crystallinity after the plasticization process. The absence of intense and well-defined peaks in all the diffractograms of the obtained films and the broad hump centered at 2θ = 19.6–19.8° underlined the severe decrease in the crystalline structure of the starch granule and the important contribution of the new amorphous phase [17,39]. These observations confirm that the glycerol used as plasticizer achieved the loss of the starch grain structure and successfully hindered the retrogradation process of the starch molecules, thereby preventing hydrogen bonding between the polymer chains [3]. Nevertheless, some small and undefined diffraction peaks centered at 2θ angles around 17, 20, 21.5, and 22° were observed, indicating that some crystallinity remained. These findings are similar to those recently published in the literature [3,8,35]. In order to study the above mentioned crystalline regions in the final films obtained, the diffractograms were deconvoluted by a Lorentzian fitting function centered on the four peaks after subtracting their baseline following the procedure described in López-Rubio et al. [40]. The results found for the XRD peaks and the widths at middle height peak are shown on Figure 7 and Table 3. The peak located at around 2θ = 17–17.4° is due to the existence of small regions of residual A-type or B-type crystallinity in the films [35,40]. On the other hand, the peaks centered at about 2θ = 19.6–19.8° and 2θ = 22.1–22.7° are characteristic of a V-type crystalline structure and appear after the starch plasticization process. This last structure consists of six-fold single helices of the amylose-glycerol complex [17]. The peak at 2θ ~ 21.5° has been described in the literature as being indicative of the dehydration of the V-type crystallinity [17]. 

Comparing the XRD spectra of corn, wheat, and rice starch films (Figure 7 and Table 3) leads us to conclude that they follow the same pattern, with small differences in the intensity and full widths at half maximum of the peaks located at 17° and 22°. It can be seen in Figure 7 that corn and wheat starch films present a similar crystallographic spectrum due to the respective starches exhibit a relatively similar structure in terms of amylose content and molecular weight. The rice starch films showed the least intense but widest peaks, which suggest that they are the most amorphous. Therefore, these results suggest that rice starch films are the most plasticized because the peak at 2θ = 17°, which is representative of the residual unplasticized starch, is less intense than that of the wheat and corn starch films, and the postplasticization peaks (2θ = 20° and 22°) are also the least defined and intense. This is probably due to rice starch having the highest molecular weight, which lead to a lower crystallization ability of the polymer, because the rearrangement of the starch chains could be hindered by its size. Similar conclusions were previously found with other polymers [41,42]. Another reason could be found in the amylose content of rice starch, which is the lowest in this study. Rindlav-Westling et al. [43] claimed that, for synthetic polymers, it is known that a linear polymer crystallizes more easily than a branched polymer based on the same monomer. The same explanation could be extrapolated to amylose, with a linear structure, and amylopectin, with a branched structure, finding that films from starches with a lower amylose content could have less crystalline regions. As will be shown later, these observations were in great consonance with the analysis of the mechanical properties. More amorphous starch films, such as rice starch films, have lower tensile strength and Young’s modulus, and higher elongation at break because of their greater mobility [3,44,45]. Similar results were reported in other studies [3,46]. 

In the case of the potato starch film (the only one obtained from a tuber), the films presented an intermediate crystalline structure between rice starch films and those made from corn and wheat starch with undefined broad peaks. This is probably due to potato starch having an intermediate molecular weight and amylose content. In addition, as it was previously mentioned, potato starch contains the highest percent of phosphate monoesters. This could also contribute to the more amorphous nature of potato starch films compared to corn and wheat starch films. Phosphate monoesters are charged groups, which can hinder the starch rearrangement during starch film preparation reducing the ordered or crystal structures of potato starch films. This corroborates the mechanical properties observed for the potato starch films, which are less resistant and more stretchable than corn and wheat starch films, and more resistant and less extensible than rice starch films. 

#### 3.2.7. Mechanical Properties

**Figure 8:**
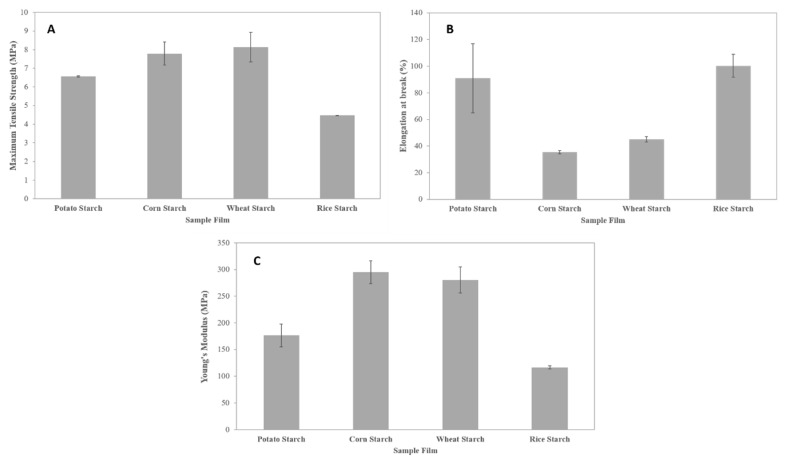
Mechanical properties of the starch films studied. (**A**) Maximum tensile strength; (**B**) elongation at break; and (**C**) Young’s modulus.

Determining the tensile properties of biodegradable films is necessary to confirm their suitability for future applications, for example, in the food packaging field. These properties can be affected by molecular weight and amylose content of starch, film thickness, polymer chain packing, chain interaction, and crystallinity of the film [10,47]. Values of the mechanical properties (maximum tensile strength, elongation at break and Young’s modulus) for the studied films are shown in Figure 8. Maximum tensile strength varied from 4.48 to 8.14 MPa, elongation at break from 35.41 to 100.34%, and Young’s modulus from 116.42 to 294.98 MPa. The differing amylose contents clearly affect the mechanical behavior of the films [48,49]. It is known that amylose forms stronger films than amylopectin [50]. As mentioned above, the amylose content found was in the following order: rice (16.9%) < potato (20.5%) < wheat (24.5%) ≈ corn (24.8%). Figure 8 shows that, in general terms, the lowest amylose content, the lowest maximum tensile strength, and stiffness but the highest film stretchability. It can be seen that wheat and corn starch films, which have similar and the highest amylose content, presented similar tensile properties and were more resistant at break and less stretchable than potato and rice starch films. These results may be due to the fact that starch films obtained from high amylose content starch usually have bigger crystalline domains [51], leading to greater mechanical resistance compared to films made from starch with a lower amylose content [38]. These crystalline domains are embedded in the amorphous matrix and could act as reinforcements for improving the film’s mechanical properties. As explained before, the film’s crystallinity is also related to the starch molecular weight. Higher starch molecular weights can hinder the crystallization process by the chain size. Therefore, the higher the amylose content and molecular weight of starch, the higher the crystallinity of the films and hence the higher the tensile strength and Young’s modulus and the lower the elongation at break of the starch film. The same conclusion was reached by Li et al. [52], Cano et al. [48], Muscat et al. [53], and Mali et al. [19]. The values obtained for the mechanical properties of the films studied in this work are similar or much better (in some cases) than those reported in previous works [8,9,10,14,38,48,54]. Films obtained from the four starch sources showed tensile properties comparable to other common polymers currently used for food packaging such as LDPE [14,55]. 

## 4. Conclusions

Starch films from four botanical sources were prepared by the casting technique. The starch granule composition and molecular weight were seen to have a significant influence on several physical and chemical properties of the films developed. The amylose content and molecular weight of the starches strongly affected the mechanical properties of the films, which were more resistant and less extensible when their amylose content and molecular weight were higher and lower, respectively. This is probably due to the fact that the films obtained from starches with a high amylose content usually present a higher quantity of crystalline domains, which are presumably responsible for improving the mechanical behavior of the TPS films. The analysis of the mechanical properties measured reveals that all the films have properties comparable to those of some conventional polymers such as LDPE, underlining the great potential of starch to replace synthetic and non-biodegradable plastics in film manufacturing.

## Figures and Tables

**Table 1:** Particle size analysis of potato, corn, wheat, and rice starches.

Starch	D(0.1) (µm)	D(0.5) (µm)	D(0.9) (µm)	Average Diameter (µm)	Span
Potato	24.50 ± 0.02	42.67 ± 0.04	72.58 ± 0.09	46.02 ± 0.05	1.127 ± 0.001
Corn	9.76 ± 0.11	15.50 ± 0.24	24.25 ± 0.92	16.38 ± 0.33	0.934 ± 0.051
Wheat	12.17 ± 0.03	19.60 ± 0.04	30.96 ± 0.06	20.75 ± 0.04	0.959 ± 0.001
Rice	5.49 ± 0.22	16.22 ± 1.56	52.32 ± 4.71	23.30 ± 2.09	2.887 ± 0.013

Results are expressed as average of three replications ± standard deviation. D(0.1), D(0.5), and D(0.9) are the particle diameters corresponding to 10%, 50%, and 90% of the cumulative distribution, respectively. Span is indicative of the particle size dispersion.

**Table 2:** Properties of the starch films obtained.

Sample Film	Thickness (mm)	Water Content (%)	Water Solubility (%)	Opacity (A_600_/mm)
Potato	0.183 ± 0.014	14.40 ± 0.34	29.74 ± 0.24	0.61 ± 0.06
Corn	0.216 ± 0.012	10.78 ± 0.68	27.88 ± 0.58	1.56 ± 0.01
Wheat	0.222 ± 0.009	12.65 ± 0.57	32.57 ± 2.04	0.93 ± 0.07
Rice	0.211 ± 0.024	12.39 ± 0.59	32.25 ± 2.74	0.90 ± 0.07

**Table 3:** 2θ peaks and their full widths at half maximum for the starch films obtained.

	Potato Starch		Corn Starch		Wheat Starch		Rice Starch	
	2θ	Width	2θ	Width	2θ	Width	2θ	Width
Peak 1	17.10	1.98	17.00	1.74	17.20	2.35	17.40	3.30
Peak 2	19.70	3.56	19.60	3.10	19.75	3.09	19.85	3.01
Peak 3	21.50	0.24	21.45	0.18	21.50	0.24	21.45	0.20
Peak 4	22.75	3.38	22.05	3.76	22.60	3.69	22.20	4.70
